# Conducting separate reviews of benefits and harms could improve systematic reviews and meta-analyses

**DOI:** 10.1186/s13643-023-02234-0

**Published:** 2023-04-15

**Authors:** Evan Mayo-Wilson, Riaz Qureshi, Tianjing Li

**Affiliations:** 1 Department of Epidemiology, UNC Gillings School of Global Public Health, Chapel Hill, NC 27599 USA; 2 Department of Ophthalmology, School of Medicine, University of Colorado Anschutz Medical Campus, Aurora, CO USA

## Abstract

Guidance for systematic reviews of interventions recommends both benefits and harms be included. Systematic reviews may reach conclusions about harms (or lack of harms) that are not true when reviews include only some relevant studies, rely on incomplete data from eligible studies, use inappropriate methods for synthesizing data, and report results selectively. Separate reviews about harms could address some of these problems, and we argue that conducting separate reviews of harms is a feasible alternative to current standards and practices. Systematic reviews of potential benefits could be organized around the use of interventions for specific health problems. Systematic reviews of potential harms could be broader, including more diverse study designs and including all people at risk of harms (who might use the same intervention to treat different health problems). Multiple reviews about benefits could refer to a single review of harms. This approach could improve the reliability, completeness, and efficiency of systematic reviews.

## Commentary

Because potential benefits and harms are important to patients and providers, guidance recommends that both types of outcomes be included in systematic reviews of interventions [1–6]. In Cochrane systematic reviews of interventions, “considering potential adverse effects” is mandatory [7]. Yet, highly cited reporting guidelines include few recommendations for reporting harms [8, 9], which are addressed in a lesser-known extension [10], and most systematic reviews report harms poorly [11, 12]. We argue that guidance for systematic reviews may overlook important differences between benefits and harms, resulting in the production of incomplete reviews and unreliable conclusions about harms. In many cases, separate systematic reviews would be preferable to combined reviews of both benefits and harms.

Harms may be described using many different terms with different and overlapping meanings, such as “adverse events” and “side effects.” [13] Information about harms might include different dimensions such as timing, duration, and severity. Here, we use “harms” broadly to refer to outcomes that negatively affect individuals receiving interventions, which may be assessed in randomized trials and other studies to investigate whether harms are causally related to interventions.

Systematic reviews including both benefits and harms can be done rigorously. For example, one review about spinal fusion included 17 randomized trials and 35 other studies [14]; using individual patient data meta-analysis, the reviewers found important differences between published and unpublished data about harms [15]. Such rigorous reviews are noteworthy because they include data that are difficult to access, apply expert knowledge of harms ascertainment, use appropriate statistical methods, and require considerable investigator time and funding.

Challenges in systematic reviews of harms arise from methods used to assess harms in primary studies [6, 16] For example, some harms can be assessed systematically like benefits (e.g., in the same way for all participants in a trial) [17]; however, many harms are assessed non-systematically in response to open-ended questions [18] or using unclear methods [19]. Because dozens or hundreds of different non-systematic harms might be identified in a single study, non-systematic harms are often reported based on study results using post hoc criteria. For example, authors might report harms that occurred in 5% or more of patients, or they might report harms occurring twice as frequently in the intervention group compared with the comparison group [20–22]. Such criteria have at least two implications for systematic reviews and meta-analyses. First, data for uncommon and rare events may be unavailable for synthesis. Second, syntheses may be biased for harms because estimates from primary studies will be included or excluded based on observed results [23, 24].

Systematic reviews of harms can compound problems arising from primary studies [25, 26]. Because many non-systematic harms might be identified in individual studies, different non-systematic harms might be identified across studies included in a systematic review. Consequently, reviewers apply selection criteria when choosing which harms to extract, synthesize, and report. For example, reviewers might choose to analyze the 10 most common harms, or reviewers might analyze harms reported in at least half of the included studies. Moreover, it is often unclear what selection criteria were applied in systematic reviews; for example, reviews often fail to define thresholds for the “most common” harms [22, 27]. Combined with trial-level selection criteria, review-level selection criteria increase the likelihood that systematic reviews will omit some harms, and that estimates for other harms will be biased. Moreover, inconsistent review-level selection criteria contribute to conflicting conclusions across reviews of the same interventions [27, 28].

Even for included studies, systematic reviews rarely use complete data about harms. Because published reports typically include less information about harms compared with benefits, unpublished reports and databases are preferred sources for information about harms in primary studies [29–31]. For example, journal articles about randomized trials typically include quantitative information about potential benefits in structured text, tables, and figures (e.g., primary and secondary outcomes). Potential benefits (“efficacy outcomes”) are defined before analysis begins, and these are often published in protocols and trial registrations. By contrast, reviewers are advised to look for other data sources about harms such as clinical study reports (CSRs), case report forms, trial registers, and individual participant data (IPD) [31–39]. Locating and reviewing multiple data sources requires more time and resources than reviewing journal articles alone [40, 41], and sometimes, trial investigators do not provide requested data, so many reviews are limited to published reports [42–44]. This is an important limitation because conclusions can change when different data sources are used, as in reviews examining the relative harms and benefits of antidepressants for young people [45, 46].

Suboptimal methods for extracting and synthesizing data also lead to incorrect results. Data extraction errors are common in meta-analyses of harms [47], especially when harms need to be coded and standardized across studies before synthesis, as is the case when primary studies do not report harms using common classification systems. For example, the Medical Dictionary for Regulatory Activities (MedDRA), Common Terminology Criteria for Adverse Events (CTCAE), and Systematized Nomenclature of Medicine Clinical Terms (SNOMED) are hierarchical systems used for recording and reporting harms in different areas, but they are not used in all primary studies or reviews [28]. Additionally, many systematic reviews do not describe plans for handling rare events in their methods [48], and many reviews deal with missing information inappropriately in their results [49]. For example, it would be informative if 80% of trials in a review failed to report the proportion of people experiencing a given harm; however, many systematic reviews omit studies with zero events from meta-analyses [50]. Common methods to handle rare events, and ignoring zero events, can lead to biased results [51–53].

Systematic reviews may come to incorrect conclusions about harms when they exclude relevant study designs. For example, systematic reviews often include only randomized trials; many reviews are designed to assess potential benefits and consider harms as secondary outcomes, if they consider harms at all [54–57]. Even systematic reviews and meta-analyses of multiple randomized trials are typically underpowered to detect uncommon and rare events [58]. Non-randomized studies can provide better estimates of differences in uncommon harms, harms that occur after prolonged exposure, harms with long latency (e.g., occurring after acute treatment or after treatment discontinuation), and harms that occur in target populations who use interventions outside of trials [59–64]. “Real-world evidence” is used increasingly to evaluate the benefis and harms of interventions, including data from electronic health records, claims, and surveillance systems [64–69]. For new drugs, pre-clinical studies also provide valuable evidence about harms that are difficult to observe in people, such as effects on developing embryos and drug interactions. For policy interventions and public health interventions, multiple studies might be needed to evaluate possible psychological harms, social harms, and effects on equity [70, 71]. Separate reviews of benefits and harms could include different types of evidence that address different types of questions about intervention effects.

There are pervasive and consequential imitations in syntheses of harms. For example, a study of cancer screening guidelines found that most guidelines failed to include information about harms. Some guidelines also compared harms associated with a single procedure with benefits that accrued through multiple procedures and treatments that resulted from cancer screening [72]. As another example, we compared systematic reviews of the drug gabapentin and found that different types of harms and different effect estimates were reported across reviews that included the same primary studies [28]. Lack of reliability is an indication that most of the reviews we assessed could be misleading.

Conclusions about harms in systematic reviews may be unreliable when reviews are limited to subsets of the at-risk population. For example, an anticonvulsant drug might be used to treat epilepsy, postherpetic neuralgia, or bipolar disorder. It might not be possible for participants with different health problems to experience the same benefits, so it would be sensible to conduct three separate reviews of: reduction in seizures for people with epilepsy, reduction in pain for people with postherpetic neuralgia, and reduction in depression for people with bipolar disorder. By contrast, participants with different health problems might be at risk of the same harms. Therefore, a comprehensive review of an intervention’s harms would include all users who are at risk of harm, regardless of the health conditions for which they use the intervention. For example, people can experience dizziness whether they take anticonvulsants to treat epilepsy, postherpetic neuralgia, or bipolar disorder. A review examining the likelihood of dizziness for people with postherpetic neuralgia should not necessarily exclude studies of people with epilepsy or bipolar disorder. When limited to studies of people with a single health problem, systematic reviews might miss relevant evidence about harms.

Overviews can combine evidence about harms across systematic reviews [73], but combining systematic reviews does not address limitations in the study types included in those reviews (e.g., randomized trials), nor can overviews resolve methodological heterogeneity across reviews (e.g., different selection criteria for reporting harms). Because it would be redundant to synthesize the same evidence about harms in different systematic reviews, we argue that only one review of harms might be needed for a given intervention.

For the reasons above, regulators including the US Food and Drug Administration (FDA) consider harms in multiple populations, animal studies, and clinical pharmacology when developing “prescribing information” for patients and clinicians [74]. Prescribing information applies to all users who might be at risk of an intervention’s potential harms. Conclusions in systematic reviews might differ from regulatory guidance when reviews are restricted to subsets of the at-risk population or when reviews are otherwise incomplete.

Conducting separate reviews about benefits and harms is a feasible alternative to the current paradigm. Multiple systematic reviews about potential benefits in different subpopulations could reference or incorporate a comprehensive review about potential harms in the entire population of users. Where applicable, heterogeneity across subpopulations could be explored in reviews of harms. This approach to systematic reviews would have implications for other research such as clinical trials and guideline development. For example, core outcome sets typically describe the minimum benefits and harms to include in all studies of a particular health problem [75]. Instead, core outcome sets for benefits could be organized around health problems (e.g., depression, anxiety), while core outcome sets for harms could be organized around different types of interventions (e.g., antidepressants, antipsychotics) [76].

In conclusion, many studies show that systematic reviews use suboptimal methods to assess harms, and that conclusions in systematic reviews are unreliable (which implies that some of their conclusions are wrong). Mandating the inclusion of harms in all systematic reviews of interventions might exacerbate rather than solve known problems; this sort of tokenism should be replaced by focused reviews of harms conducted by teams with appropriate expertise. Reviewing harms and benefits separately could minimize redundant work across reviews of the same interventions, and it could reduce the likelihood that reviews of the same interventions reach inconsistent (incorrect) conclusions.

## Data Availability

Not applicable.
